# Demyelinating polyneuropathy combined with brachial plexopathy after nivolumab therapy for hodgkin lymphoma: a case report

**DOI:** 10.1186/s12883-023-03177-8

**Published:** 2023-03-30

**Authors:** Chulmin Park, Kyoung Tae Kim

**Affiliations:** Department of Rehabilitation Medicine, Keimyung University Dongsan Hospital, Keimyung University School of Medicine, 1095 Dalgubeol-daero, Dalseo-gu, Daegu, 42601 Republic of Korea

**Keywords:** Nivolumab, Demyelinating peripheral polyneuropathy, Hodgkin lymphoma

## Abstract

**Background:**

Nivolumab is an immune checkpoint inhibitor that targets the programmed cell death-1 protein and is effective in treating advanced cancer. However, it is also associated with various immune-related neurological complications, including myasthenia gravis, Guillain–Barré syndrome, and demyelinating polyneuropathy. These complications can easily mimic other neurological diseases and have greatly varying therapeutic approaches depending on the underlying pathophysiology.

**Case presentation:**

Here, we report a case of nivolumab-induced demyelinating peripheral polyneuropathy involving the brachial plexus in a patient with Hodgkin lymphoma. Approximately 7 months after nivolumab treatment, the patient experienced muscle weakness with a tightness and tingling sensation in the right forearm. Electrodiagnostic studies showed features of demyelinating peripheral neuropathy with right brachial plexopathy. Magnetic resonance imaging revealed thickening with a diffuse enhancement of both brachial plexuses. The patient was eventually diagnosed with nivolumab-induced demyelinating polyneuropathy involving the brachial plexus. Oral steroid therapy improved motor weakness and sensory abnormalities without aggravation.

**Conclusion:**

Our study indicates the possibility of nivolumab-induced neuropathies in cases involving muscle weakness with sensory abnormalities of the upper extremity following nivolumab administration in patients with advanced cancer. Comprehensive electrodiagnostic studies and magnetic resonance imaging are helpful in the differential diagnosis of other neurological diseases. Appropriate diagnostic and therapeutic approaches may prevent further neurological deterioration.

## Background

Tumor cells have various evasion mechanisms to escape the immune system. Immune checkpoints, namely programmed cell death-1 (PD-1) and cytotoxic T lymphocyte antigen-4 (CTLA-4), and their immunologic pathways are well-known immune systems in the human body. They are expressed on T cells and induce T-cell inhibition by binding to ligands on tumor cells [1, 2].

Nivolumab is an immune checkpoint inhibitor (ICI) that targets the PD-1 protein, leading to the blockage of the inhibitory immune checkpoint pathways in T cells [3]. Nivolumab has been used to effectively manage advanced cancers with abundant expression of ligands of PD-1 on their tumor cell surfaces, such as relapsed Hodgkin lymphoma (HL), non-small-cell lung cancer, and metastatic melanoma, because of this distinct mechanism of action [4–6]. In particular, nivolumab is one of the important therapeutic options in refractory HL, given that the disease has 10–30% of reported recurrence rate [7]. However, it is also associated with various immune-related neurological complications, including myasthenia gravis, Guillain–Barré syndrome, and demyelinating polyneuropathy [8, 9]. When compared to other immune-related complications of nivolumab (e.g. gastrointestinal disorders such as colitis with 63.4% of incidences) these neurological adverse events are relatively uncommon with an estimated incidence of 6.1–12% [10–12]. But it is important to consider such possibilities in patients with advanced cancer who receive the treatment in that the symptoms can easily mimic those of other direct tumor invasions or autoimmune-related neuropathies, and can lead to inevitable postponement or suspension of the chemotherapy, even causing fatal conditions such as respiratory failure [3]. Moreover, the therapeutic approaches vary greatly depending on the pathophysiology of the disease. Here, we report a case of nivolumab-induced demyelinating peripheral polyneuropathy involving brachial plexopathy in a patient with refractory HL.

## Case presentation

A 32-year-old woman presented to our outpatient clinic with ongoing muscle weakness accompanied by a tingling sensation and tightness in the right forearm. She had been diagnosed with HL 8 years ago. She started the first chemotherapy right after the diagnosis and received six treatments every 3 weeks. Specifically, she was administered oral prednisone (100 mg), intravenous brentuximab (1.8 mg/ kg), cyclophosphamide (750 mg/m^2^), doxorubicin (50 mg/m^2^), and vincristine (1.4 mg/m^2^). She also had autologous hematopoietic stem cell transplantation 2 months after the chemotherapy. Unfortunately, the lymphoma recurred as multiple metastases 9 months after the first line of treatment. Therefore, the patient received additional chemotherapy with nivolumab alone. She was scheduled to receive 30 intravenous injections of nivolumab (3 mg/kg) every 2 weeks. However, because of relapses in her lymphoma despite the nivolumab treatment, the salvage nivolumab treatment was also administered. Muscle weakness with a tightness and tingling sensation in the right forearm developed after the 17th injection, approximately 7 months initiating salvage treatment. The manual muscle test (MMT) score was grade 3 for right elbow flexion, elbow extension, finger flexion, finger extension, and finger abduction.

The patient reported tightness from her right elbow to her fingertips, while the tingling sensation in the right arm correlated with the dermatome of the ulnar nerve. She also experienced difficulties in daily living, such as writing and using spoons and chopsticks.

**Fig. 1:**
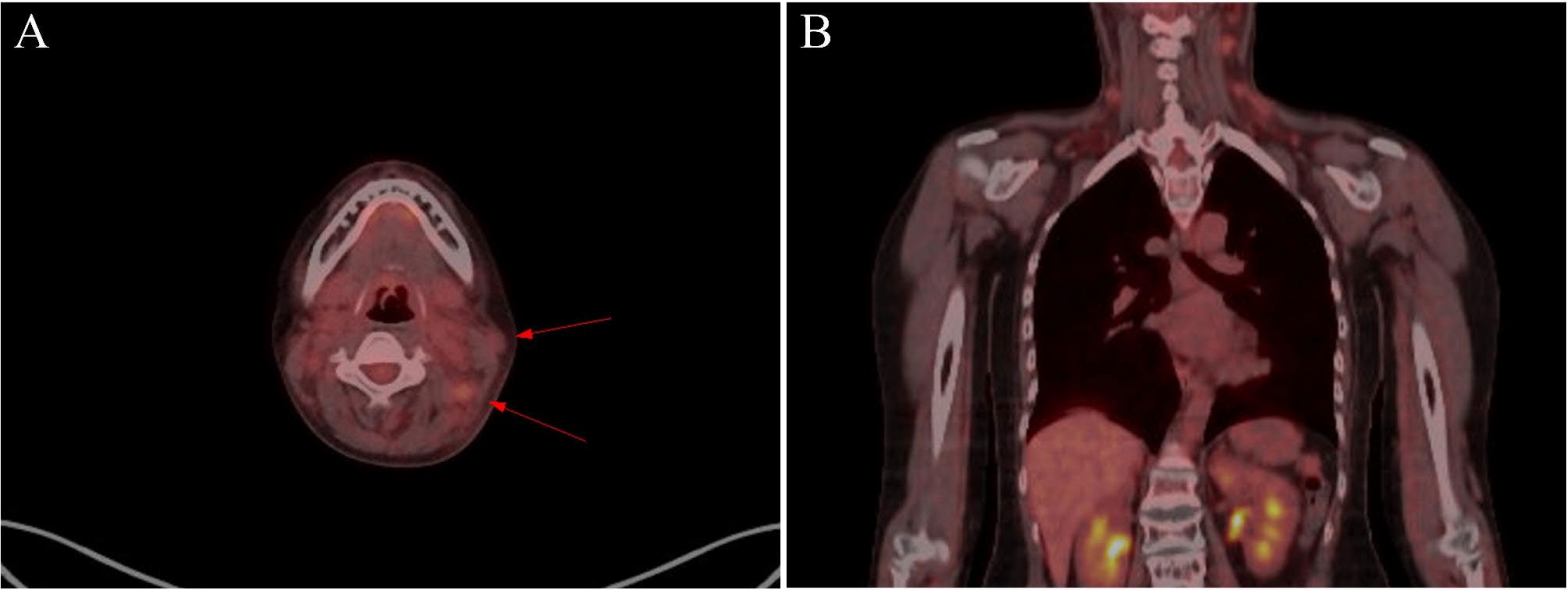
Positron emission tomography scan images. (A) The axial image shows intense uptake in the left cervical lymph nodes, consistent with the recurrence of lymphoma. Arrow: cervical lymph nodes. (B) The coronal image shows no direct invasion of the lymphoma along nerve segments, including those of the brachial plexus

**Fig. 2:**
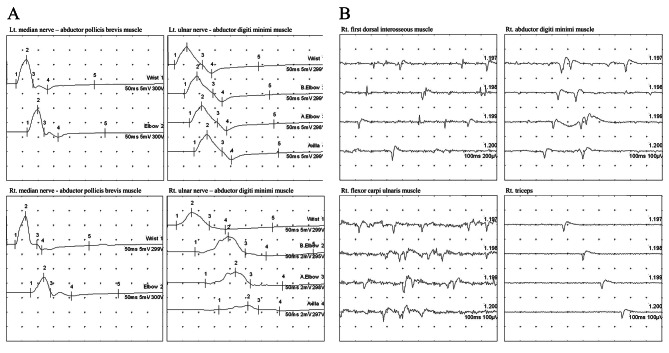
Results of electrodiagnostic studies. (A) Motor NCS reveals conduction block and temporal dispersion in the right median and ulnar nerves and reduced CMAP amplitude in the right ulnar nerve. (B) Needle EMG shows denervation potentials in the right FDI, ADM, FCU, and triceps muscles NCS: Nerve conduction study; EMG: Electromyography; CMAP: Compound muscle action potential; Lt.: Left; Rt.: Right; APB: Abductor pollicis brevis; FDI: First dorsal interosseus; ADM: Abductor digiti minimi; FCU: Flexor carpi ulnaris

We conducted an F-18 fluorodeoxyglucose positron emission tomography (FDG-PET) scan to check for direct cancer invasion to the nerves in her right upper extremity. The scanning results revealed intense uptake in the left cervical lymph nodes and newly formed hypermetabolic lesions in the right pleura and right retrocrural area, consistent with lymphoma recurrence; however, no evidence of direct tumor invasion in the right brachial plexus or peripheral nerves was detected (Fig. 1). Next, we performed electrodiagnostic studies, including nerve conduction study (NCS) and needle electromyography (EMG), to identify neurologic deficits. Sensory NCS showed reduced sensory nerve action potential amplitudes in the right medial and lateral antecubital cutaneous nerves. Motor NCS demonstrated demyelinating features with conduction block in the right ulnar and median nerves and reduced compound muscle action potential amplitude in the right ulnar nerve (Table 1; Fig. 2A).

**Table 1:** Motor NCS findings showing reduced CMAP amplitude in the right ulnar nerve and conduction block in the right median and ulnar nerves

Nerve/Sites	Latency (ms)	Amplitude (mV)	Conduction Velocity (m/s)
Left median nerve (abductor pollicis brevis muscle)			
Wrist	2.95	8.4	
Elbow	6.45	7.8	57.1
Right median nerve (abductor pollicis brevis muscle)			
Wrist	2.85	9.9	
Elbow	7.75	5.4	**44.9**
Left ulnar nerve (abductor digiti minimi muscle)			
Wrist	2.20	6.2	
Below the elbow	5.50	6.1	54.5
Above the elbow	6.90	5.8	64.3
Axilla	8.45	5.9	58.1
Right ulnar nerve (abductor digiti minimi muscle)			
Wrist	3.00	**4.6**	
Below the elbow	9.00	**2.1**	**30.0**
Above the elbow	12.25	**1.6**	**30.8**
Axilla	16.45	**0.5**	**21.4**

(NCS: nerve conduction study; CMAP: compound muscle action potential)

Needle EMG indicated abnormal spontaneous activity in the right first dorsal interosseous, abductor digiti minimi, triceps, and flexor carpi ulnaris muscle (Fig. 2B). Therefore, the electrodiagnostic studies revealed demyelinating-type peripheral neuropathy with right brachial plexopathy, mainly involving the middle and lower trunks.

**Fig. 3:**
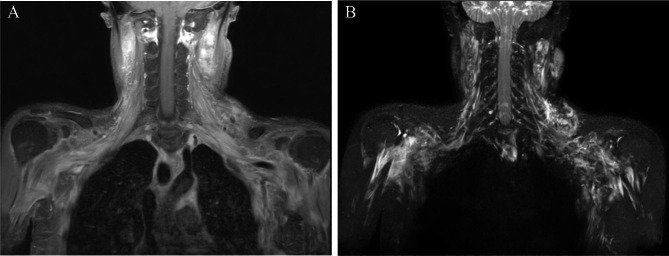
Brachial plexus magnetic resonance images show thickening with a diffuse enhancement of the entire segments of both brachial plexuses. (A) T1- and (B) T2-weighted magnetic resonance images of the brachial plexuses show thickening with a diffuse enhancement of the entire segments of both brachial plexuses

Additionally, brachial plexus magnetic resonance imaging (MRI) with gadolinium enhancement showed thickening with diffuse enhancement of all segments of both brachial plexuses and the peripheral nerves without direct invasion or recurrence of the tumor; this implied brachial plexopathy, including inflammatory conditions such as autoimmune- or chemotherapy-induced neuropathies (Fig. 3).

Laboratory studies, including sera and cerebrospinal fluid (CSF) analysis, showed no specific abnormal findings or evidence of other autoimmune-related polyneuropathies. Specifically, the studies showed no elevated levels of anti-ds-DNA antibodies (Ab) immunoglobulin (Ig) A, Ig E, Ig M, complement 3, complement 4, rheumatoid factors, anti-streptolysin O Ab, antinuclear Ab, anti-glomerular basement membrane Ab, anti-Jo-1 Ab, anti-myeloperoxidase Ab, anti-proteinase-3 Ab, anti-Ro Ab, Anti-La Ab, and lupus anticoagulants in the patient’s sera. The CSF analysis showed normal results of protein and cell count. In addition, there were no abnormal Ig G oligoclonal bands and elevated levels of Ig G.

We diagnosed nivolumab-induced demyelinating polyneuropathy combined with bilateral brachial plexopathy based on the patient’s medical history and evaluation results. Consequently, the patient received intravenous methylprednisolone for 5 days (1000 mg/day), followed by oral prednisolone (40 mg/day, tapered by 10 mg weekly). Her sensory symptoms showed improvement immediately after treatment. The patient reported partial recovery of the muscle weakness and tingling sensation in the right forearm at 1 month after steroid administration. The MMT score was grade 5 for right elbow flexion, right elbow extension, and right finger flexion. A slight improvement was also observed in right finger abduction and extension, and the oral prednisolone dose was eventually tapered to 5 mg every alternate day. However, the patient continued the oral steroid regimen because her muscle tightness and sensory abnormalities redeveloped after discontinuing the administration. The regular administration of oral prednisolone prevented further exacerbation of the symptoms until 4 months since the steroid therapy initiation.

## Discussion

ICIs are monoclonal antibodies that bind to specific checkpoint molecules along the immunological pathway, such as CTLA4 and PD-1 [13]. PD-1 is a cell surface receptor expressed on the T cell surface, and its signaling cascade facilitates the auto-regulatory function of T cells [13]. Nivolumab, a monoclonal antibody that binds to PD-1, acts as an inhibitor of this protein and causes anti-cancer immune responses [14]. It is the first FDA-approved ICI in relapsed HL as a third-line drug. Growing evidence supports its administration as a second or first-line drug in newly diagnosed HL [15]. In a recent retrospective study, nivolumab showed comparable safety and efficacy in treating patients with refractory HL [16]. However, it is known for its various immune-related complications. Although the mechanism is poorly understood, the T cell activation and simultaneous reduction in the suppression of autoantibody-producing B cells by nivolumab result in an autoantibody attack on the host’s normal tissues [9]. De Grado et al. recently suggested that elevated serum autoantibodies, autoreactive cytotoxic T cells, increased inflammatory cytokine levels, and complement activation can cause immune-related complications [17]. These immune-related adverse events include colitis, endocrine dysfunction, and inflammatory neuropathies [14, 18]. Among them, neurological adverse events are an increasingly recognized complication of ICIs targeting PD-1, with a reported incidence of up to 12% [12].

Several cases of nivolumab-induced neurological complications have been reported. Nukui et al. reported a case of nivolumab-induced acute multiple cranial neuropathies and demyelinating polyradiculoneuropathy, in which the symptoms and NCS findings mimicked Guillain–Barré syndrome [3]. Alhammad et al. described two cases of nivolumab-induced brachial plexus neuritis [19], where nivolumab-induced brachial plexopathy had been reported as a predominantly lower trunk disease with high signal intensity on T2-weighted MRI. Furthermore, Bover et al. noted nivolumab-induced transverse myelitis in a patient with metastatic melanoma. The patient showed progressive intentional tremors on both the upper and lower limbs and marked improvement of neurological symptoms with intravenous methylprednisolone [20].

In our case, the patient had progressive muscle weakness and sensory abnormalities in the right arm. As the patient was diagnosed with HL with multiple metastases, we excluded other cancer-related neuropathies, including the initial exclusion of direct invasion of metastatic cancer. FDG-PET scanning showed no hypermetabolism in the right arm or axillary area.

NCS and needle EMG findings showed demyelination features in the right arm’s nerves. The conduction velocity was decreased in the right ulnar (30.0 m/s) and the median nerve (44.9 m/s), and the waveform showed significant temporal dispersion. All these findings together implied demyelinating brachial plexopathy. Fukumoto et al. described a case of acute demyelinating polyneuropathy induced by nivolumab. The NCS findings were consistent with those of our patient, showing prolonged distal latency and reduced conduction velocity on the median and ulnar nerves [18].

Interestingly, MRI showed diffuse enhancement of the entire segments of the bilateral brachial plexus and the peripheral nerves. In contrast, the symptoms and abnormal findings of the electrodiagnostic studies were observed only in the right upper extremity. Therefore, considering that the patient’s first neurological symptoms appeared 7 months after nivolumab therapy, we diagnosed nivolumab-induced demyelinating polyneuropathy combined with bilateral brachial plexopathy.

According to recent studies and the clinical practice guidelines of the American Society of Clinical Oncology, discontinuing nivolumab and administering corticosteroids is the mainstay of treatment in general. Intravenous immunoglobulin or plasmapheresis therapy is considered if the effect of corticosteroid therapy is insufficient or the patients present with severe neurological complications such as weakness limiting walking or respiratory failure [11, 17, 21]. In our case, the patient’s symptoms were relieved after intravenous and oral steroid pulse therapy, and were maintained for 4 months without aggravation.

To the best of our knowledge, this is the first report of nivolumab-induced demyelinating peripheral neuropathy combined with bilateral brachial plexopathy involving all segments of both brachial plexuses. Interestingly, a remarkable discrepancy between the image results and the patient’s clinical symptoms made it difficult to diagnose accurately. However, additional brachial plexus MRI with enhancement, FDG-PET, and detailed electrodiagnostic evaluations helped make the diagnosis.

## Conclusions

Our study demonstrates that when muscle weakness with sensory symptoms of the upper extremity occurs after nivolumab administration in patients with advanced cancer, the possibility of nivolumab-induced neuropathies should be considered. Appropriate diagnostic and therapeutic approaches are important because these neuropathies can mimic other neuropathies caused by various etiologies and even lead to postponement or suspension of planned chemotherapy. Thorough electrodiagnostic tests and imaging studies, including MRI and FDG-PET, benefit in making differential diagnosis. Timely management, including intravenous corticosteroids, can prevent further neurological deterioration.

## Data Availability

All data used and analyzed during the current study are available from the corresponding author upon reasonable request.

## Abbreviations

CTLA-4: Cytotoxic T lymphocyte antigen-4
PD-1: Programmed cell death-1
ICI: Immune checkpoint inhibitor
HL: Hodgkin lymphoma
MMT: Manual muscle test
FDG-PET: F-18 fluorodeoxyglucose positron emission tomography
MRI: Magnetic resonance imaging
NCS: Nerve conduction study
CMAP: Compound muscle action potential
EMG: Needle electromyography
CSF: Cerebrospinal fluid
Ab: Antibodies
Ig: Immunoglobulin
